# Thrombospondin and VEGF-R: Is There a Correlation in Inflammatory Bowel Disease?

**DOI:** 10.1155/2013/908259

**Published:** 2013-07-18

**Authors:** Jaroslaw Wejman, Michal Pyzlak, Dariusz Szukiewicz, Dorota Jarosz, Wieslaw Tarnowski, Grzegorz Szewczyk

**Affiliations:** ^1^Department of Pathology, Professor Witold Orlowski Public Clinical Hospital, Medical Center for Postgraduate Education, Czerniakowska 231, 00-416 Warsaw, Poland; ^2^Department of General & Experimental Pathology, Medical University of Warsaw, Krakowskie Przedmiescie 26/28, 00-927 Warsaw, Poland; ^3^Department of Gastroenterology and Hepatology, Medical Center for Postgraduate Education, Roentgena 5, 02-781 Warsaw, Poland; ^4^Department of Surgery, Professor Witold Orlowski Public Clinical Hospital, Medical Center for Postgraduate Education, Czerniakowska 231, 00-416 Warsaw, Poland

## Abstract

Up to date several authors discussed interactions between cells forming inflammatory infiltrates in the course of inflammatory bowel disease (IBD), mainly dealing with endoscopic biopsy specimens. These usually contain only mucosa. We have evaluated full bowel wall sections, which seems to be especially important in patients with Crohn's disease (CD). The purpose of our study was to evaluate the relationship between vascular density and expression of thrombospondin-1 (TSP-1) and vascular endothelial growth factor receptor 1 (VEGFR-1) in full-thickness tissue fragments of intestinal wall taken from patients after colectomy, comparing those with IBD to non-IBD control group. Histological sections were immunostained with antibodies against CD-31, TSP-1, and VEGFR-1 and analyzed by pathologists with the use of computer-assisted morphometrics. Our research showed significantly higher vascular density and vascular area percentage in all layers of bowel wall in patients with CD when compared to control. We have also demonstrated differences in vascular density distribution between ulcerative colitis (CU) and CD and between CU and control. However we have not found statistically significant correlation between those findings and VEGFR-1 or TSP-1 expression. Our results might suggest existence of different, TSP-1 independent pathways of antiangiogenesis in IBD.

## 1. Introduction

Ulcerative colitis (CU) and Crohn's disease (CD) are known as chronic inflammatory bowel diseases (IBD). There is still some controversy about IBD etiology. Among factors associated with inflammatory bowel disease, angiogenesis plays important role in development of clinical symptoms. It has been shown that exacerbation of IBD increases angiogenesis especially in ulcerative colitis [1, 2]. This finding produced a concept of antiangiogenic therapy in IBD, as presented by Danese et al. [3].

It was reported that anti-angiogenesis in the development of IBD can be measured by detection of thrombospondin [4, 5]. Since anti-angiogenesis is almost always associated with angiogenesis, numerous angiogenic factors have been indicated as possible counterparts to thrombospondin. Among them, vascular endothelial growth factor (VEGF) is thought to play a crucial role by stimulating migration and proliferation of endothelial cells (ECs) and the expression of angiogenesis-related genes. There are 7 types of VEGF family and VEGF-A with two receptors VEGFR-1 and VEGFR-2 that are most important in angiogenesis [6, 7]. Thrombospondin (TSP) is a 450 kD adhesive glycoprotein that was initially discovered in platelet *α*-granule [8]. Thrombospondin is one of the extracellular matrix adhesive molecules, including also laminin, fibronectin, fibrinogen, and von Willebrand factor. Thrombospondins are secreted glycoproteins that modulate cell-matrix interactions, influence platelet aggregation, and support neutrophil chemotaxis and adhesion [9]. Thrombospondin-1 (TSP-1) is known as an antiangiogenic factor. There are several articles about the role of TSP-1 in IBD, most of these based upon animal models [4, 5].

The purpose of our study was to assess angiogenesis in correlation with expression of VEGFR-1 and thrombospondin-1 to see if they mediate in maintaining balance between angiogenesis and anti-angiogenesis in the course of IBD. We have decided to use computer-assisted morphometrics to facilitate the analysis of vascular density and immunohistochemically highlighted expression of angiogenic factors.

## 2. Materials and Methods

We have analyzed full-thickness intestinal wall histological sections routinely prepared out of formalin fixed, paraffin embedded tissue samples. All samples were collected after colectomy in the course of IBD. All our IBD-affected intestines were resected because of an active phase of inflammation. CU bowels were presented with grade 5 of the disease, according to grading scale for histological assessment of inflammation in ulcerative colitis by Geboes et al. [10]. Intestines with CD have showed a severe phase of the disease according to Geboes [11]. Patients in the control group underwent bowel removal due to noninflammatory, nonvascular conditions. All tissue specimens were collected during standard grossing procedures. The number of patients in each group was shown in Table 1.

Only one tissue fragment containing full thickness of intestinal wall was collected from each patient. In IBD groups tissue fragments were collected from sites involved by the disease. The control group was formed with the use of fragments that were collected from macroscopically unchanged bowel wall. All tissue samples were fixed in formalin, embedded in paraffin, and sectioned into 4 micrometer sections, according to standard histopathology protocol.

**Figure 1:**
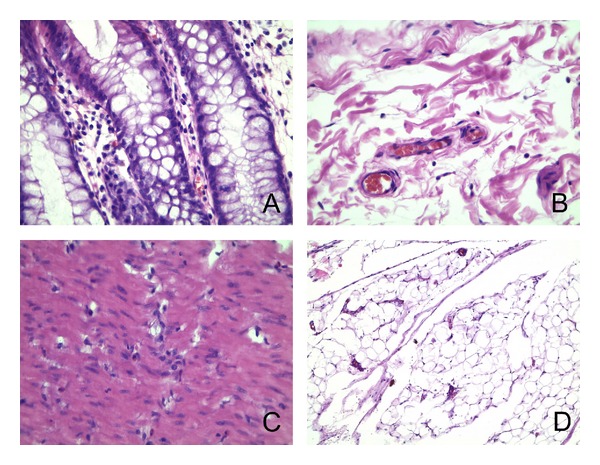
Microphotographs from hematoxylin & eosin stained sections of normal large bowel wall. A: mucosa (400x); B: submucosa (400x); C: muscularis (400x); D: subserosa (100x).

**Figure 2:**
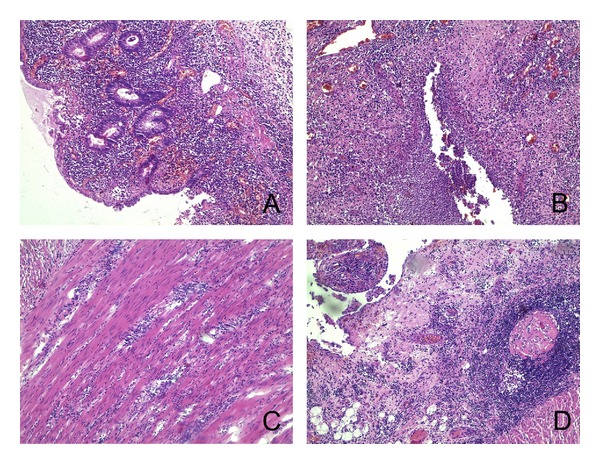
Microphotographs from hematoxylin & eosin stained sections of bowel wall from a patient with Crohn's disease. A: partially ulcerated mucosa (100x); B: fissure-like ulcer penetrating from mucosa into submucosa (100x); C: inflammatory changes in muscularis propria (100x); D: Inflammatory changes in subserosa (100x).

**Figure 3:**
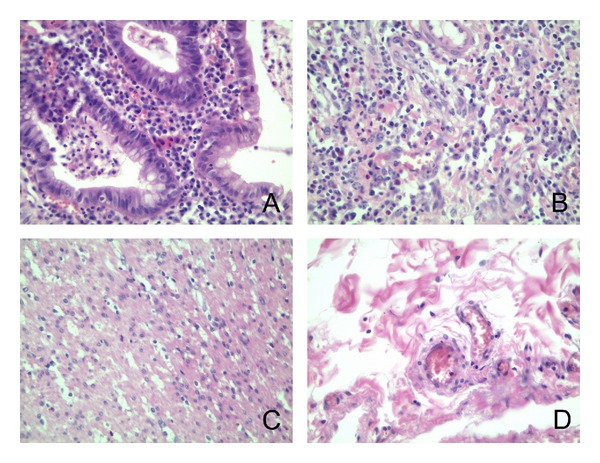
Microphotographs from hematoxylin & eosin stained sections of bowel wall from a patient with ulcerative colitis. A: inflammatory changes within mucosa (400x); B: inflammatory changes in submucosa (400x); C: muscularis (400x); D: subserosa (400x).

One section in each case was stained routinely with hematoxylin & eosin to evaluate the extent of the disease or to confirm the lack of pathology (in the control group) (see Figures 1, 2, and 3).

Other sections were immunostained with antibodies against CD-31 (to highlight vessel walls), thrombospondin-1 and VEGFR-1 (to show the expression of both). Antibodies and their respective dilutions are shown in Table 2. Visualization was performed with En-Vision Flex visualization system (Dako). All antibodies were earlier validated for routine use with immunohistochemistry in our facility.

Immunostained sections were subsequently photographed and analyzed.

For morphometric analysis we have used Leica Quantimet workstation along with ImageJ custom macros [12–14].

**Figure 4:**
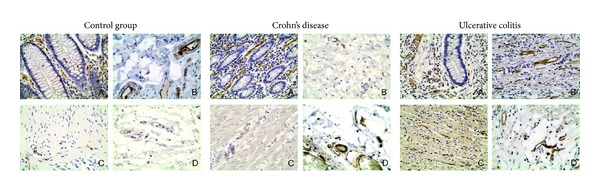
Immunohistochemical expression of VEGFR-1 (magnification 400x). A: mucosa; B: submucosa; C: muscularis; D: subserosa.

Each layer of vessel wall was analyzed separately. Vascular density was evaluated based upon two parameters—immunostaining against CD31 per square mm and percentage of section area covered by vessels. Thrombospondin expression was independently evaluated by two pathologists on a semiquantitative manner. VEGFR-1 expression was evaluated by counting the vessels that showed immunohistochemical reaction in endothelial cells. Immunohistochemical expression of VEGFR-1 has been shown in Figure 4. 

Statistical analysis of the results (Mann-Whitney *U* test, Kruskall-Wallis test, correlation analysis) was performed with the use of *R* programming language [15]. 

## 3. Results

**Figure 5:**
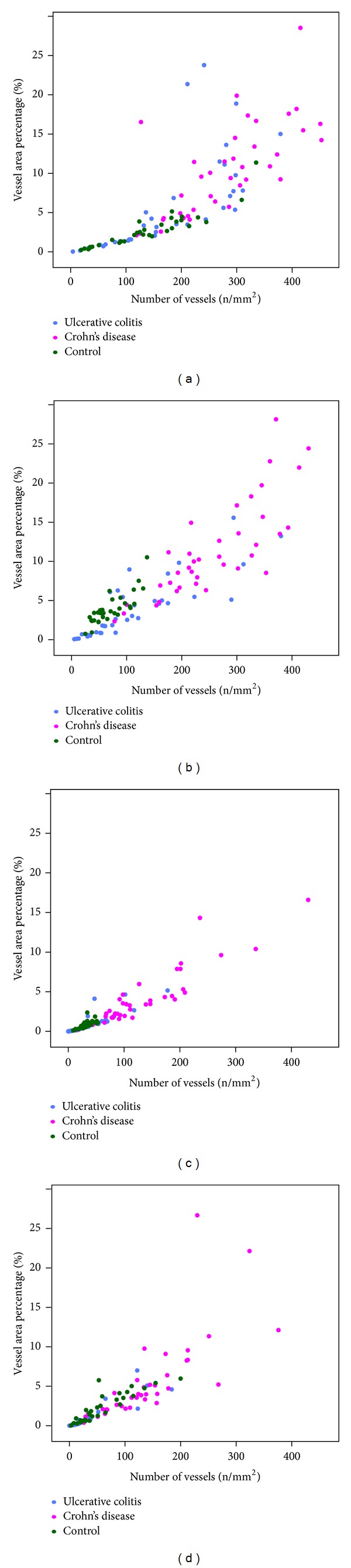
Number of vessels versus vessel area percentage in intestinal wall layers. (a) Mucosa, (b) submucosa, (c) muscularis propria, and (d) subserosa.

**Figure 6:**
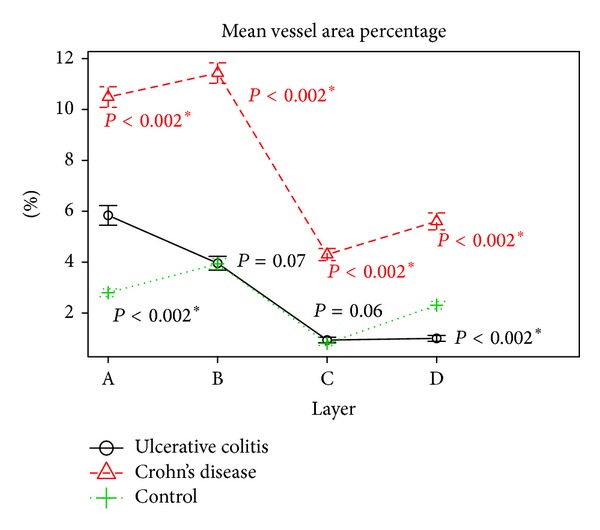
Mean vessel area percentage in bowel wall layers by group. A: mucosa; B: submucosa; C: muscularis; D: subserosa statistically significant values were marked with (∗).

**Figure 7:**
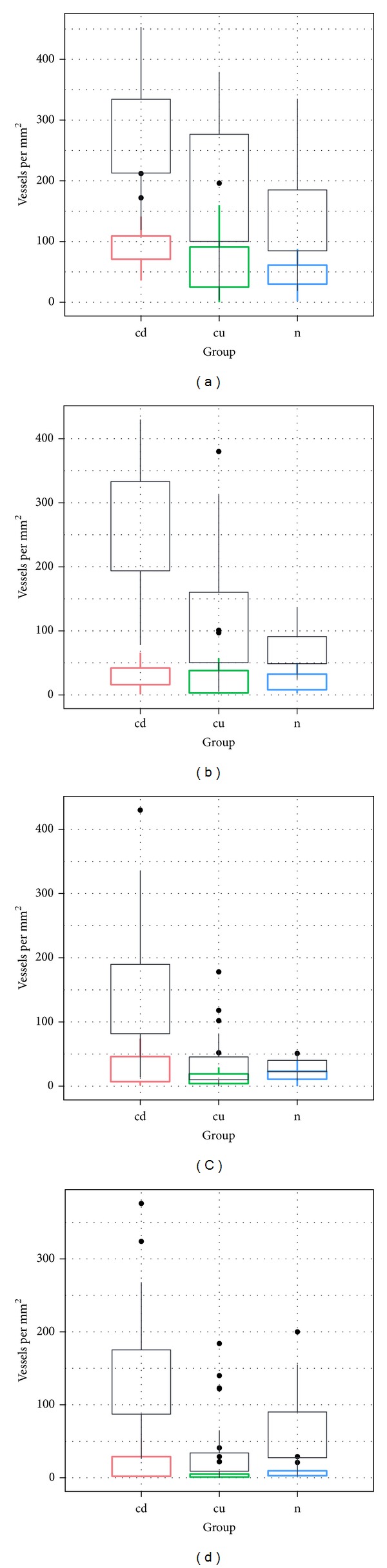
Comparison of total number of vessels (black) and number of VEGFR-1 positive vessels (color). (a) Mucosa, (b) submucosa, (c) muscularis propria, and (d) subserosa. cd: Crohn disease, cu: ulcerative colitis, and n: control.

Figures 5–7 show vascular density values in tissue layers of bowel wall in our study. 

**Figure 8:**
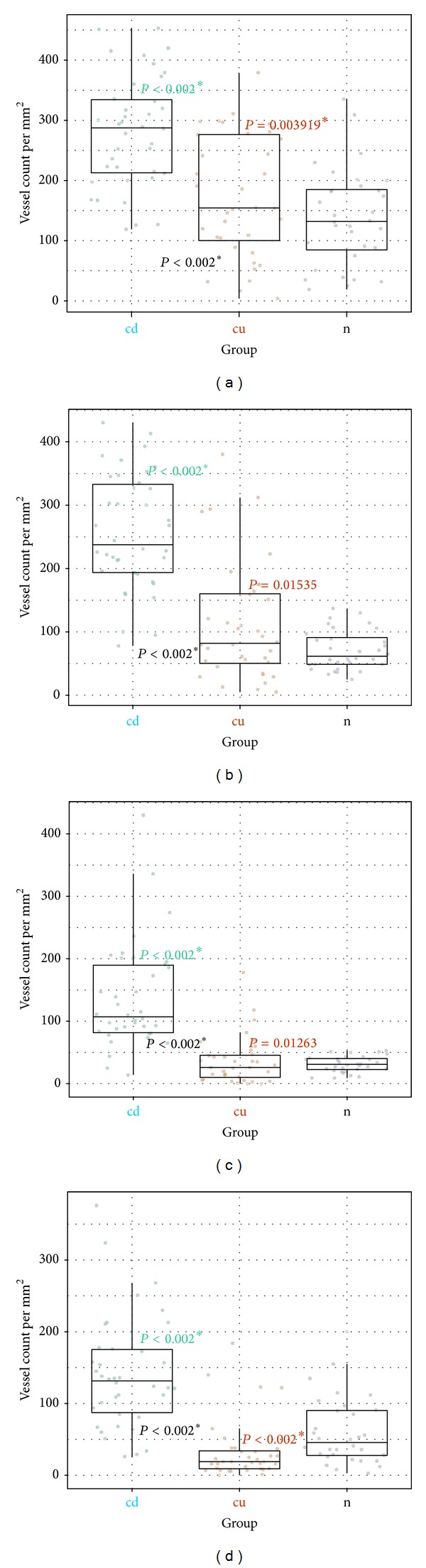
Vessel count differences between groups by bowel wall layer. cd: Crohn's disease; cu: ulcerative colitis; n: control. (a) Mucosa; (b) submucosa; (c) muscularis; (d) subserosastatistically significant differences were marked with (∗). *P* values: color: cd/cu versus control; grey: cd versus cu.

We found statistically significant differences in both mean vessel count and mean vessel area fraction in Crohn's disease when compared to normal bowel and ulcerative colitis in comparison to normal bowel (see Table 3, Figures 6 and 8).

Differences in vascular area percentage between ulcerative colitis and control were insignificant for submucosa and muscularis propria. It is interesting, that our results showed significantly higher vascular density in subserosa of control group patients when compared to ulcerative colitis as shown in Figures 6 and 8.

We have also shown significant differences in both mean vessel count and mean vessel area fraction throughout the all layers between Crohn's disease and ulcerative colitis (*P* < 0.002).

Our study showed no statistically significant correlation between IBD type and VEGFR-1 (Flt1) expression and between vascular density and VEGFR-1 expression (see Table 4 and Figure 7). 

**Figure 9:**
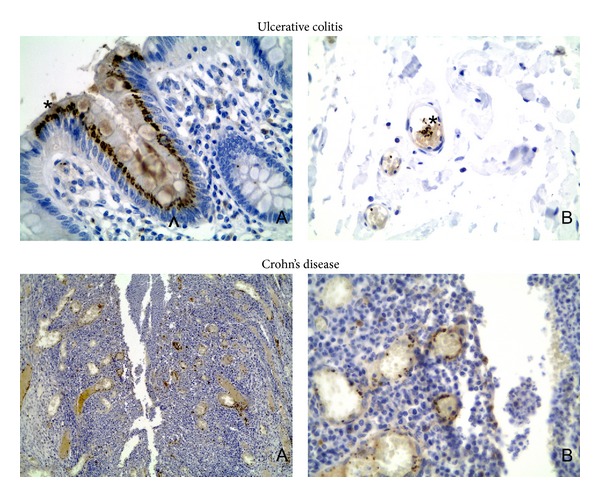
Immunohistochemical expression of TSP-1. Ulcerative colitis: A: TSP-1 expression within surface (∗) and crypt (arrowhead) epithelium (400x); B: expression of TSP-1 in thrombocyteswithin subserosal blood vessel (∗). Crohn's disease: expression of thrombospondin in thrombocytes within vessels surrounding fissure-like ulcer. A: low magnification (100x), B: detailed view (400x).

TSP-1 was sporadically found (4 cases, expression within intestinal epithelium, see Figure 9) only in samples from patients with IBD (3 in CU and 1 in CD), and it seems to be rather artifactual and insufficient to make any statistical correlation between TSP-1 expression and vascular density or VEGFR-1 expression. We have observed strong internal immunohistochemical control reaction in thrombocytes within blood vessels. Answering the title question, we could not find a strict correlation between thrombospondin and vascular endothelial growth factor receptor expression. 

## 4. Discussion

The results of our study support macro- and microscopic pattern in cases with active phase of inflammatory bowel disease—intensive blood vessels congestion. We have also revealed significantly increased number of blood vessels in almost all of the four layers of bowel wall while comparing Crohn's disease and ulcerative colitis with normal bowel wall. Staining with CD31 was shown to be of great importance not only allowing the assessment of the number of vessels but also showing blood vessels essentially dilated. Increased vessel count could support the fact of angiogenesis accompanied by VEGF. However, in our opinion, Flt1 (VEGFR1) is not a marker of angiogenesis—this study not revealed statistically significant reaction in normal and IBD bowels. This is contrary to Konno et al. who stated that expression of Flt-1 (among others) was increased in active CU [16]. We believe that a better marker of angiogenesis could be the expression of another VEGF receptor-VEGFR2 (KDR/Flk-1), which was also studied by the same authors. Importance of VEGFR2 in angiogenesis was also described by Ferrara et al. [6] and Usui et al. [17]. We share the opinion of Scaldaferri et al. who stated that, after the induction of colitis, the expression of both VEGF-A and VEGFR-2 was markedly enhanced, whereas no increase in the expression of VEGFR-1 was observed [18]. Only four positive thrombospondin expression findings in our research could be explained by several mechanisms. All our cases with IBD were collected from patients with acute and severe phases of the diseases. This could explain inflammation-induced changes in TSP-1 expression. Also, increased stimulation of angiogenic pathways could completely suppress anti-angiogenesis (inhibition of thrombospondin expression). It is also possible that the pathways of anti-angiogenesis in IBD rely on mediators other than TSP-1. We also cannot exclude that our antibody reacted with different epitopes (nonspecific for that of TSP-1) leading to artifactual staining. Our findings did not confirm previous reports on the possible role of TSP-1. Zak et al. and Punekar et al. [4, 5] have studied TSP-1-deficient mice with experimental colitis and suggested that TSP-1 might decrease angiogenesis. Alkim et al. studied only mucosal samples and found that the expression of TSP-1 was higher in IBD groups relative to healthy control group [19]. We could not find such a correlation because TSP-1 tissue reaction was sporadic and weak. We would like to emphasize that we studied the full thickness of bowel wall in contrast to most reports dealing with samples from the intestinal mucosa only. According to our findings, the number of blood vessels increased, and they became dilated in all layers of bowel wall (especially in the course of Crohn's disease) showing that angiogenesis is one of the main pathological processes occurring not only within mucosa but also within other layers of bowel wall.

## Figures and Tables

**Table 1:** Groups of patients in the study.

Group	Number of patients (*N*)
Control	32
Crohn's disease	38
Ulcerative colitis	36

**Table 2:** Antibodies used for immunostaining.

Antibody	Clone	Manufacturer	Dilution	Epitope retrieval
Monoclonal mouse anti-human CD31	Endothelial cell, Clone JC70A	Dako	Ready-to-use (manufacturer prediluted)	HIER, pH = 9.0
Monoclonal mouse anti-human thrombospondin	Clone TSP-B7	Sigma-Aldrich	1 : 100	HIER, pH = 6.0
Rabbit anti-VEGFR-1 antibody	Polyclonal	Sigma-Aldrich	1 : 20	HIER, pH = 6.0

HIER: heat induced epitope retrieval.

**Table 3:** Mean vessel count differences [vessel count/mm^2^ (SD)]. Comparison of Crohn's disease versus control, ulcerative colitis versus control, and ulcerative colitis versus Crohn's disease.

Layer	Control n/mm^2^	CD n/mm^2^	CU n/mm^2^
Mucosa	171.10 (99.36)	346.43 (113.50)^∗#^	215.98 (125.26)^#^
Submucosa	87.58 (38.42)	318.06 (112.34)^∗#^	138.82 (114.96)^#^
Muscularis	38.35 (15.83)	168.29 (104.71)^∗#^	44.55 (46.51)^#^
Subserosa	74.00 (57.66)	174.11 (96.84)^∗#^	41.53 (52.11)^∗#^

Mean number of vessels/mm^2^. Numbers in parentheses show standard deviation.

*Significant differences between CD or CU and Control; ^#^Significant differences between CD and CU.

CD: Crohn's disease, CU: ulcerative colitis.

**Table 4:** Vessel count and VEGFR-1 positive vessel count correlation analysis (Spearman's rho).

	Control	Crohn's disease	Ulcerative colitis
Mucosa	*R* Spearman = −0.01399 *P* = 0.9590	*R* Spearman = 0.1156 *P* = 0.1156	*R* Spearman = 0.5004 *P* = 0.0484
Submucosa	*R* Spearman = 0.1864 *P* = 0.4893	*R* Spearman = −0.03824 *P* = 0.8802	*R* Spearman = 0.02432 *P* = 0.9288
Muscularis	*R* Spearman = 0.06038 *P* = 0.8242	*R* Spearman = −0.05168 *P* = 0.8386	*R* Spearman = 0.2426 *P* = 0.3652
Subserosa	*R* Spearman = 0.1167 *P* = 0.6669	*R* Spearman = −0.04359 *P* = 0.8636	*R* Spearman = 0.1828 *P* = 0.4981
